# Dioscorea bulbifera polysaccharide and cyclophosphamide combination enhances anti-cervical cancer effect and attenuates immunosuppression and oxidative stress in mice

**DOI:** 10.1038/srep19185

**Published:** 2016-01-12

**Authors:** Hongxia Cui, Ting Li, Liping Wang, Yan Su, Cory J. Xian

**Affiliations:** 1 College of Environmental and Chemical Engineering, Yanshan University, Qinhuangdao, Hebei 066004, China; 2 Hebei Province Key Laboratory of Applied Chemistry, Qinhuangdao, Hebei 066004, China; 3 Sansom Institute for Health Research and School of Pharmacy and Medical Sciences, University of South Australia, Adelaide, SA 5001, Australia

## Abstract

Cyclophosphamide (CTX) is commonly used in cancer chemotherapy, which causes immunosuppression and tissue oxidative stress at high doses. As potential protective agents, some polysaccharides were shown to have anti-tumor, anti-inflammatory and/or anti-oxidant properties. This study explored potential effects of oral treatment of *Dioscorea bulbifera* polysaccharides (DBLP at 100 or 150 mg/kg) in U14 cervical tumor-bearing mice treated with CTX (25 mg/kg). While CTX suppressed tumor growth (65.4% inhibition) and DBLP alone also inhibited tumor (25.6% at 100 mg/kg or 37.6% at 150 mg/kg), CTX+DBLP combination produced tumor inhibition rates of 5.6 (for 100 mg/kg DBLP) or 9% (for 150 mg/kg) higher than CTX alone. While tumor itself and CTX treatment reduced thymus and/or spleen/body weight indices, DBLP alone or CTX + DBLP combination attenuated this reduction. DBLP lowered peripheral blood T-cell subpopulation CD^4+^/CD^8+^ ratio, and DBLP+CTX combination attenuated CTX effect in lifting CD^4+^/CD^8+^ ratio. Tumor itself and CTX treatment heightened oxidative stress (with decreased superoxide dismutase but increased lactate dehydrogenase and malondialdehyde levels in serum and tissues), which was attenuated by DBLP treatment, and DBLP+CTX combination suppressed CTX-induced oxidative stress. Combination use of DBLP with CTX can potentially enhance CTX anti-tumor effect and can attenuate CTX-induced immunosuppression and oxidative stress in U14 cervical tumor-bearing mice.

Cervical cancer is a common female cancer, which has over 500,000 new cases diagnosed every year globally^1^. Despite the availability of low-cost and effective methods to prevent cervical cancer, it still kills thousands of women worldwide each year, most of whom are in low-income countries^2^. Although there has been continuing research for more effective and better treatments, surgery, radiotherapy, and chemotherapy are the most commonly used modalities in cervical cancer treatment. While fertility-sparing surgical treatment is only applicable to the young patients and the patients in the early cancer stage^3^, radiotherapy and chemotherapy unfortunately often cause many side effects, such as organ toxicity and immunotoxicity^4^.

Cyclophosphamide (CTX) is a commonly used chemotherapeutic drug. As a DNA alkylating agent, its active metabolite can kill cancer cells^5^,^6^,^7^ and can also have a negative immunomodulatory effect. The effect of CTX on T cells is known to be complex and dose-dependant, and T cell lymphopenia and immunosuppression can develop at its higher doses^8^,^9^. In addition, CTX generates free radicals and therefore causes oxidative stress in tissues and cells^10^. Therefore, supplementary treatments that could enhance CTX antitumor effect and attenuate its adverse effects would be of significant importance and will lessen the side effects of high-dose CTX treatment^11^.

To enhance the anti-cancer efficacy and reduce the toxicity of CTX, recently, there have been various studies investigating potential adjuvant treatments that can prevent/attenuate the adverse effects. Polysaccharides are the major bioactive substances in medinal herbs^12^. Many studies have reported that combining polysaccharides with chemotherapy might enhance the anti-tumor response, reduce the toxicity of chemotherapy, and improve quality of life^13^,^14^. Some polysaccharides are known to possess various biological activities, including immunomodulation, anti-viral, anti-tumor, anti-oxidation, and anti-inflammation^15^. Among these biological activities, the immunomodulatory effect of some polysaccharides is most remarkable^16^. Many studies have reported that some polysaccharides are ideal immune-enhancing agents, as they can improve host defense against pathogens and modulate adaptive immunity^17^. Furthermore, previous studies have reported that many kinds of polysaccharides have potential and/or potent capabilities to prevent oxidative damages in living tissues due to their ability in scavenging free radicals, and they can protect normal tissues against oxidative stress^18^,^19^. *Dioscorea bulbifera* has been used as a traditional Chinese medicine for thousands of years. However, it remains to be explored whether the polysaccharides of *Dioscorea bulbifera* can be used to prevent side effects of CTX chemotherapy.

The anti-cervical cancer effect of *Dioscorea bulbifera* polysaccharides in mice has been preliminarily shown in our previous study^20^. In the current study, as a step to investigate whether the combined use of crude *Dioscorea bulbifera* polysaccharides (DBLP) and cyclophosphamide (CTX) could be useful in cervical cancer treatment, we explored the effect of *Dioscorea bulbifera* polysaccharides plus CTX combination in treating cervical cancer in tumor-bearing mice. We hypothesized that *Dioscorea bulbifera* polysaccharides could attenuate CTX treatment-induced immunosuppression and oxidation, and to improve the anti-tumor effect of CTX.

## Results

### Treatment effects on animal status and movement

In this study, treatment effects on appearances, behaviours and weight gains of animals were examined, which were found not significantly affected in all treatment groups except for the negative control. Animals could move about freely. Their furs looked smooth and normal, and their food intakes and water consumption appeared also normal.

Treatment effects on changes in body weights were shown in Table 1. By the end of the experiment, while the normal blank control mice had a 38.1% increase in weights, mice in the negative control group (tumor group without any treatments) had the least growth (17.7% increase, P < 0.01 compared to all other groups), which could be due to the tumor’s influence. This was followed by the CTX alone group (positive control, 24.6% increase) which could be due to the toxicity of CTX that is known to damage some normal tissues including gut mucosa, bone and bone marrow^21^. However, weight gains of mice in the other groups did not show obvious differences when compared to the blank controls. The weight gains of mice with polysaccharide alone treatment markedly improved when compared to the negative control group. In the two groups treated with CTX plus polysaccharides, the weight gains were almost the same as that of the blank control group, which could be due to the enhanced antitumor effect and reduced toxicity of CTX to normal tissues when it was used in combination with polysaccharides. These findings suggest that the nutritional state and general wellbeing of the animals were the best in the combination treatment group and were the worst in the negative control group.

### Treatment effects on tumor growth

Effects of CTX treatment with or without low or high dose of DBLP on the tumor inhibition rate were analyzed 15 days after tumor cell inoculation (Table 2). When there was no drug treatment (negative control group), the tumor was grown to around 3.3 g. CTX alone treatment (positive control group) significantly suppressed tumor growth (65.26% inhibition; P < 0.01 vs negative control). Compared to the negative control, DBLP alone treatment also inhibited tumor growth (25.68% inhibition for low dose and 37.46% for high dose, both at P < 0.01). Compared with the CTX alone group, the tumor inhibition rates of the combination groups showed trends of being higher despite the lack of statistically significant differences, being 5.74% higher in CTX + low dose group and 9.06% higher in CTX + high dose group than that in CTX alone group. These indicate that the effect of DBLP plus CTX is slightly better than CTX treatment alone in suppressing tumor growth, suggesting that effect of CTX in tumor inhibition may be potentially enhanced by DBLP.

### Treatment effects on thymus and spleen gland/body weight indices

**Figure 1:**
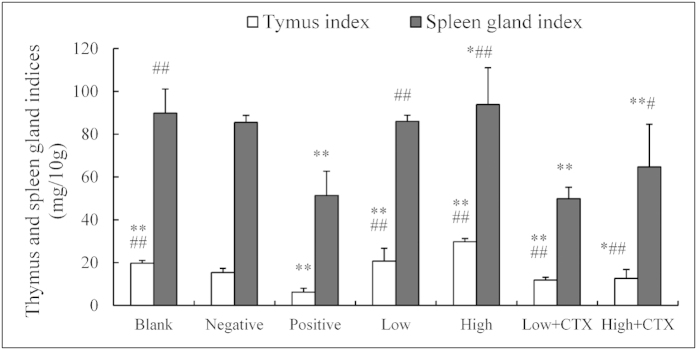
Effects of CTX treatment with or without DBLP on thymus and spleen gland/body weight indices in tumor-bearing mice. Blank, blank control; Negative, negative control; Positive, positive control; Low, low dose DBLP group; High, high dose DBLP group; Low + CTX, low dose DBLP group + CTX; High + CTX, high dose DBLP group + CTX. Data were expressed as mean ± SD of 10 mice. *P < 0.05 and **P < 0.01 compared to negative control, #P < 0.05 and ##P < 0.01 compared to positive control.

To examine potential immunomodulatory effects of CTX treatment with or without DBLP, the thymus and spleen/body weight indexes of treated animals were analyzed (Fig. 1). It can be seen that, when compared with the no-tumor blank control group, the thymus index in negative control group (tumor-bearing without drug treatment) was lower (P < 0.01) and the spleen gland index showed trends of being lower despite a lack of statistically significant differences. This suggests that tumor causes a decline in the immunity of the mice. Compared with the negative control group, the thymus index and spleen gland index were lower in positive control group (P < 0.01) and higher in the DBLP alone groups (P < 0.01). These findings indicated that immunosuppression could be caused by CTX and that DBLP had an immunomodulatory effect. The thymus index in the combination groups were higher than that in the positive control group (P < 0.01), and the spleen gland index in the CTX plus high dose DBLP combination group was higher than that in the positive control group (P < 0.05). These results indicate that DBLP may potentially overcome the immunosuppressive action of CTX to a certain extent.

### Treatment effects on contents of CD^4+^ and CD^8+^ T-cells in peripheral blood

**Figure 2:**
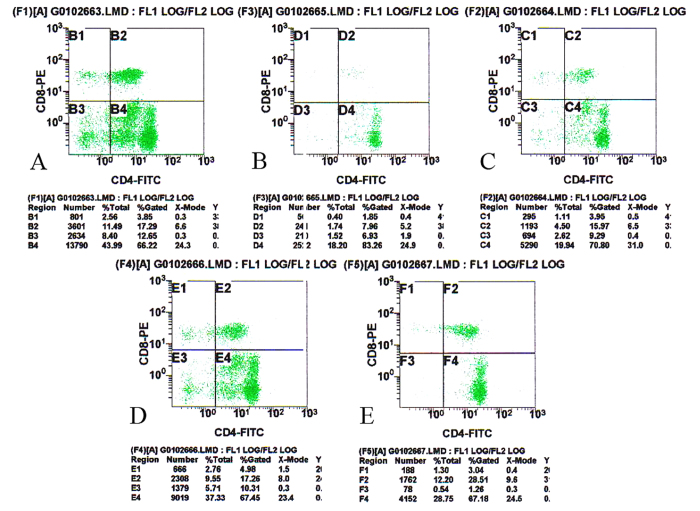
Replesentative graphs of flow cytometry analyses on effects of CTX treatment with or without DBLP on CD^4+^ and CD^8+^ T-cell contents in peripheral blood of tumor-bearing mice ((**A**) blank control; (**B**) positive control; (**C**) negative control; (**D**) high dose DBLP; (**E**) high dose DBLP + CTX).

The immunomodulatory effects of CTX treatment with or without DBLP were further examined on the cellular level. Peripheral blood CD^4+^ and CD^8+^ T-cell contents were analysed by flow cytometry (Fig. 2) and data are presented in Table 3. Compared with the blank control group, the CD^4+^/CD^8+^ value in negative control group was unchanged, suggesting that tumor bearing did not affect ratio of T-cell subpopulations. The CD^4+^/CD^8+^ value after CTX alone treatment (positive control group) had an increased trend when compared with the negative control group, as its CD^4+^ gated % was increased from 70.80 to 83.26 and CD^8+^ gated % decreased from 3.95 to 1.85 (Fig. 2 and Table 3). After DBLP alone treatment (DBLP group), the CD^4+^/CD^8+^ value was lower than the negative control group, with its CD^4+^ gated % being decreased from 70.80 to 67.45 and CD^8+^ gated % increased from 3.95 to 4.98. After the combination treatment, the CD^4+^/CD^8+^ value tended to be higher than that in the negative control group and was lower than that in the positive control group.

### Treatment effects on serum and tissue total superoxide dismutase (T-SOD) activities

**Figure 3:**
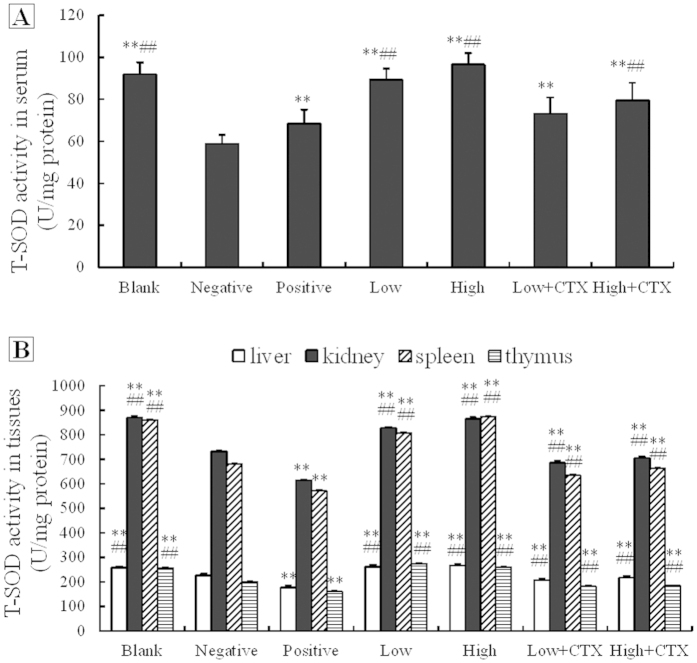
Effects of CTX treatment with or without DBLP on serum (**A**) and tissue (**B**) total superoxide dismutase (T-SOD) activities in tumor-bearing mice. Blank, blank control; Negative, negative control; Positive, positive control; Low, low dose DBLP group; High, high dose DBLP group; Low + CTX, low dose DBLP group + CTX; High + CTX, high dose DBLP group + CTX.Data were expressed as mean ± SD of 10 mices. **P < 0.01 compared to negative control, and ##P < 0.01 compared to positive control.

To examine potential oxidative stress-modulatory effects of CTX treatment with or without DBLP following tumor cell inoculation, activity levels of the total superoxide dismutase (T-SOD, an antioxidant enzyme) in the serum (Fig. 3A) and in the thymus, spleen gland, liver and kidney (Fig. 3B) were analyzed in treated animals. Tumor growth alone (negative control group) decreased the T-SOD activity when compared with the blank control group (P < 0.01) (Fig. 3A). Following CTX (positive control) or DBLP treatment, the serum T-SOD level was increased when compared with the negative control group (P < 0.01). The T-SOD activity in the CTX and high dose DBLP combination treatment group was higher than that in the CTX alone positive control group (P < 0.01).

As shown in Fig. 3B, tumor growth alone (negative control group) decreased the T-SOD activities in all tissues examined when compared with the blank control group (P < 0.01). Following CTX (positive control) treatment, the T-SOD activity was lower than that in the negative control group (P < 0.01). Compared with the positive control group, the T-SOD activity increased in the combination treatment groups (P < 0.01). These data demonstrated that SOD activity in serum or in various tissues could be reduced by the tumor growth in mice, and that it can be further reduced by CTX treatment in tumor-bearing mice. However, this reduction could be slowed down by DBLP treatment and the combined use of DBLP and CTX can increase the T-SOD levels when compared to CTX-alone treatment in tumor-bearing mice.

### Treatment effects on serum LDH activity and MDA content

**Figure 4:**
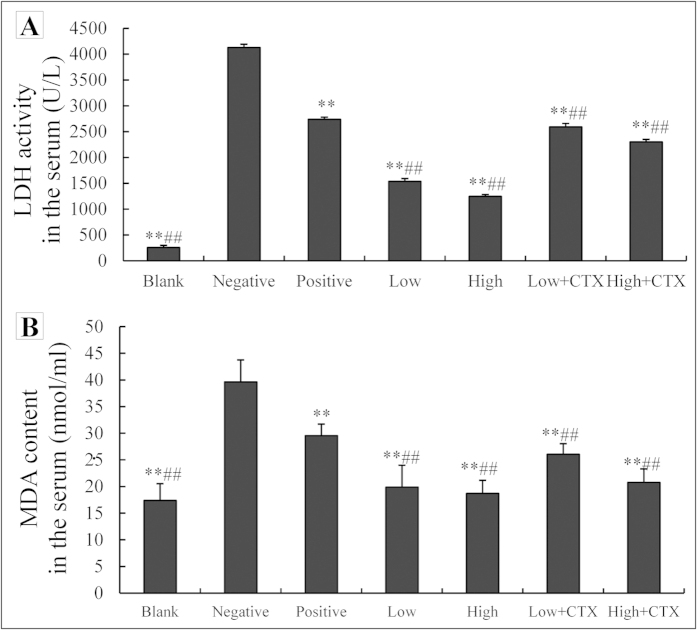
Effects of CTX treatment with or without DBLP on serum lactic acid dehydrogenase (LDH) activity (**A**) and malondialdehyde (MDA) content (**B**) in tumor-bearing mice. Blank, blank control; Negative, negative control; Positive, positive control; Low, low dose DBLP group; High, high dose DBLP group; Low + CTX, low dose DBLP group + CTX; High + CTX, high dose DBLP group + CTX. Data were expressed as mean ± SD of 10 mices. **P < 0.01 compared to negative control, and ##P < 0.01 compared to positive control.

To further examine modulatory effects of treatment with CTX with or without DBLP on tissue damage and oxidative stress, serum activity of lactic acid dehydrogenase (LDH, a marker of normal tissue damage) and contents of malondialdehyde (MDA, a lipid peroxidation product) were analyzed in treated animals (Fig. 4). Tumor cell inoculation and tumor growth (negative control group) significantly increased the serum LDH activity (Fig. 4A) and MDA content (Fig. 4B) when compared with the blank control group (16.1 folds for LDH and 2.3 folds for MDA, P < 0.01). LDH activity of CTX treatment alone (positive control) and DBLP alone treatment all decreased when compared with the negative control group (P < 0.01). Similarly, CTX treatment alone (positive control) and DBLP alone treatment also reduced serum MDA content when compared with the negative control group (P < 0.01). The LDH activities in the combination treatment groups were lower than that in the positive control group (P < 0.01). Similarly, the serum MDA contents in the combination treated groups were lower than that in the positive control group (P < 0.01).

## Discussion

Cyclophosphamide (CTX) is a commonly used drug for cancer chemotherapy (eg for cervical cancer) and its high doses are known to cause severe lymphopenia and immunosuppression and oxidative stress to normal tissues in cancer patients. In our recent study, we observed an anti-cervical cancer property of *Dioscorea bulbifera* polysaccharides (DBLP) in mice^20^. In the current study, we explored the treatment benefit effects of adjuvant treatment with DBLP in tumor-bearing mice. We observed that combined use of DBLP and CTX showed a better anti-tumor effect, and that tumor and/or CTX-induced immunosuppression and oxidative stress could be ameliorated by DBLP in tumor-bearing mice.

Previously, polysaccharides isolated from Alchornea cordifolia had been found to possess an anti-tumor effect^15^. Similarly, in our recent study, DBLP has been shown to have an anti-tumor property in mice bearing U14 cervical carcinoma^20^. In the current study, we observed that DBLP treatment alone also inhibited growth of U14 cervical tumor dose-dependantly (25.6% inhibition for low dose and 37.6% for high dose). In addition, DBLP and CTX combination treatment showed a trend of having a higher ability in inhibiting tumor growth than CTX treatment alone, suggesting that the effect of CTX in inhibiting tumor could potentially be strengthened by DBLP supplementation.

Our data demonstrated that U14 solid tumor growth in mice causes a decline in the immunity as shown particularly by a significantly lower thymus index, and that CTX treatment can further cause immunosuppression (lower thymus index and spleen gland index). The thymus is an important immune organ, being the central hematopoietic site for making T cells, which are major players of the adaptive immune system in vertebrates^22^. The spleen is the largest secondary immune organ in the body, and it plays an important role in maintaining immune homeostasis^23^,^24^. Suggesting its positive immunomodulatory effect, our data showed that DBLP can attenuate the immunosuppressive effect caused by tumor growth or by the combination of tumor growth and CTX chemotherapy. Chen *et al.*^25^ and Wang *et al.*^26^ obtained a similar tendency of changes in thymus index and spleen gland index as in our study when they respectively studied polysaccharides from Polygoni Multiflori Radix Praeparata and Strongylocentrotus nudus eggs.

The current study has also examined treatment effects on peripheral blood T-cell subpopulation ratio. Interestingly, while tumor bearing alone was shown not to affect CD^4+^/CD^8+^ value, CTX treatment significantly increased the CD^4+^/CD^8+^ value in the turmor-bearing mice. Furthermore, DBLP treatment was found to lower the CD^4+^/CD^8+^ value in the turmor-bearing mice, and DBLP+CTX combination treatment significantly attenuated CTX effect in lifting the CD^4+^/CD^8+^ value. While the CD^4+^/CD^8+^ value as a estimation of overall immunity status is still being debated (with some literatures suggesting lower CD^4+^/CD^8+^ values representing better immunity^27^, and other literatures suggesting the contrary^28^), our results indicated that a lower CD^4+^/CD^8+^ value may represent a better overall immunity status in this tumor bearing and CTX chemotherapy setting. Our results suggest that immunosuppression can be caused by CTX chemotherapy, which can be attenuated to a certain extent by DBLP supplementary treatment in tumor-bearing mice.

Increased oxidative stress, representing an imbalance between intracellular production of free radicals and the cellular defence mechanisms^29^, has long been recognized to play an important role in cancers and in cancer treatment-induced adverse effects^30^. To confirm the anti-oxidative damage action mechanism of DBLP in inhibiting tumor growth and countering CTX chemotherapy-induced adverse effects, the current study has investigated effects of U14 solid tumor growth and CTX chemotherapy with/without DBLP treatment on levels of 3 molecules related to oxidative stress, namely superoxide dismutase (SOD), lactic acid dehydrogenase (LDH), and malondialdehyde (MDA). SOD is an enzyme that plays an important role in maintaining the balance of oxidation and antioxidation and thus protecting cells from oxidative stress by removing superoxide anion free radical (O^2−^) of aerobic metabolism. LDH is normally an important enzyme of energy metabolism in the body, and an increase of LDH content has been predominantly seen in malignant disease which can lead to serious damage to normal cells^31^, and recently it has been found that its inhibition induces oxidative stress and suppresses tumor progression^32^. Malondialdehyde (MDA) is one secondary product of lipid peroxidation and has been extensively studied as a potential biomarker for oxidative stress^33^. Here, our analyses showed that U14 solid tumor growth can significantly induce oxidative stress in mice as it causes a reduction of SOD activity in serum and various tissues but increases in serum LDH activity and MDA content. CTX chemotherapy further intensifies oxidative stress in tumor-bearing mice as it furthers reduces serum/tissue SOD activity (which is consistent with a previous report that CTX can decrease activity of SOD of liver^34^), increases serum LDH activity (which has been associated with more serious toxicity of CTX^35^), and elevates serum MDA contents (which is consistent with a previous finding of increased levels of MDA following CTX treatment^32^).

However, in tumor-bearing mice, the reduction of SOD levels was shown to be slowed down by DBLP treatment, and the combined use of DBLP and CTX increased the SOD levels when compared to CTX-alone treatment. Consistent with our findings, Chen *et al.*^25^ obtained a similar tendency of changes in SOD activity in the serum of mice treated with a polysaccharide from Polygoni Multiflori Radix Praeparata. Similarly, Yu Q. *et al.* observed the same tendancy of treatment effects of Ganoderma atrum polysaccharide on liver and kidney SOD levels in mice^36^. Furthermore, the current study observed that the serum LDH activity and serum MDA content in tumor-bearing mice can be decreased by DBLP treatment alone, and that CTX and DBLP combination treatment (particularly with the high dose DBLP) can reduce tumor-induced LDH activity and MDA content to a greater extent than the treatment with CTX alone. Previously, Liu *et al.*^37^ found that serum LDH activity in mice could be decreased by treatment with the polysaccharides extracted from Zizyphus jujube cv. Huanghetanzao, and this finding was partly consistent with our data on DBLP effects on serum LDH activity. Similarly, it was previously demonstrated that CTX-induced increased MDA content in mice could be relieved by treatments with polysaccharides derived from Polygoni Multiflori Radix Praeparata and polysaccharides from G. lucidum^25^,^38^, and here our results show a similar tendency of treatment effects on serum MDA contents with treatment with DBLP. Taken together, data of the current study suggest that lipid oxidation of serum and tissues in tumor-bearing mice can be induced by tumor growth itself and by CTX chemotherapy, and that this oxidative stress can be attenuated by DBLP treatment. Furthermore, combined use of DBLP and CTX can attenuate serum and tissue oxidation induced by CTX treatment.

While various previous studies have demonstrated biological activies of polysaccharides, findings from the current study also suggest that polysaccharides extracted were active ingredients responsible for the anti-tumor effect seen in our study. In this study, amounts of extract given to the animals (per body weights) and the anti-turmor effects observed were all based on the amounts of polysaccharides given. In addition, in the current study, polysaccharides were extracted from *Dioscorea bulbifera* of 4 different batches, and the resulting polysaccharide extracts all consistently showed an anti-tumor activity. Consistency of the anti-tumor effect of different batches of polysaccharides extracted was also reported recently^20^.

In conclusion, the combined use of DBLP and CTX displays a better anti-tumor effect than DBLP or CTX treatment alone in tumor-bearing mice. BDLP treatment can increase thymus and spleen gland indices, and DBLP and CTX combination treatment can improve the CD^4+^/CD^8+^ T-cell ratio when compared to CTX treatment alone. Furthermore, compared to CTX treatment alone, the combination treatment can increase the SOD activity in serum and tissues and reduce serum LDH activity and MDA content. This study suggests that combination use of DBLP with CTX can enhance the anti-tumor effect of CTX and can ameliorate the immunosuppression and lipid oxidation side effects caused by CTX in tumor-bearing mice.

## Materials and Methods

### Reagents

*Dioscorea bulbifera* L. was purchased from Tangren Drugstore of Qinhuangdao in China. Crude polysaccharides of *Dioscorea bulbifera* L. (DBLP) were extracted as described previously 20, and were composed of mannose (Man 1.8%), galactose (Gal 18.4%), xylose (Xyl 1.2%), arabinose (Ara 0.7%), glucose (Glu 40.7%), galacturonic acid (GalA 35.8%), and glucoronic acid (GluA 1.4%). Cyclophosphamide (CTX) was obtained from Shanxi Powerdone Pharmacuetics Co (Lot number 1008421; Shanxi, China) and diluted to 25 mg/ml in distilled water before use. Mouse cervical cancer U14 cells were purchased from Tumor Cell Bank of Chinese Academy of Medical Sciences (Beijing, China). Female Kunming mice of about 18 g were purchased from Experimental Animal Center of Chinese Academy of Military Medical Sciences (Beijing, China). Lactic acid dehydrogenase (LDH) kit, total superoxide dismutase (T-SOD) kit, and malondialdehyde (MDA) kit were all purchased from Nanjing Jiangcheng Science and Technology Co (Nanjing, China). Anti-mouse CD^4+^ and anti-mouse CD^8+^ monoclonal antibodies were obtained from Pharmingen (San Diego, CA). All other chemicals used were of analytical grade.

### Animal model and treatments

Adult female Kun Ming specific pathogen free mice (6 weeks old, about 18 g) were divided into seven groups randomly, which were blank control, no drug treatment negative control, CTX alone positive control, low dose DBLP alone, high dose DBLP alone, low dose DBLP+CTX, and high dose DBLP+CTX, respectively (Table 4) (n = 10/group). The blank control group mice (group 1) were not inoculated with tumor cells, while the other six groups (groups 2–7) received subcutaneous injection with U14 tumor cells (0.2 mL of 2 × 10^7^ cells/mouse, to the oxter of the right fore limb). This was taken as day 0 and the experimental treatment started 24 h later, with groups receiving water or DBLP gavage or CTX intraperitoneal injection (0.2 ml, 25 mg/kg body weight) (Table 4). This work was approved by the insititutional animal ethics committee (Yanshan University, Hebei, China), and the methods were carried out in accordance with the approved guidelines.

### The status and body weights of mice

Food intake, water consumption, furs, and movement of mice were monitored during the experiments. Body weights were measured initially and 24 hours after the last DBLP dose (or fifteen days following tumor cell inoculation).

### Tumor, thymus, and spleen sizes

Fifteen days later and 24 hours after the last dose, all the mice were humanely killed. Tumor block, thymus and spleen glands were dissected and weighed, and then tumor inhibition rate (%), thymus index and spleen gland index were calculated. The tumor inhibition rate (%) was calculated as [(Average tumor weight of control group – Average tumor weight of drug group)/Average tumor weight of control group] × 100. The thymus or spleen gland index was calculated as (Thymus or spleen gland weight/Body weight without tumor) × 10.

### CD^4+^ and CD^8+^ T-cell contents

By the end of the experiment on the 16th day, peripheral blood of all groups was collected for examination of treatment effects on CD^4+^ and CD^8+^ T-cell contents immediately after the mice were killed. Peripheral blood mononuclear cells were isolated by centrifugation and were stained with FITC-conjugated anti-mouse CD^4+^ and PE-conjugated anti-mouse CD^8+^ antibodies. Cells were analyzed in an Epics-XLII flow cytometer, gated properly and a total of 10000 events were acquired and analyzed using Expo 32 ADC Analysis Software.

### Oxidative stress

For examining treatment effects on oxidative stress, by the end of the experiment on the 16th day, sera were obtained from peripheral blood smaples. The total superoxide dismutase (T-SOD) activity in serum was detected using the T-SOD kit as instructed. In addition, liver, kidney, thymus, and spleen gland were collected and homogenized for measuring the SOD activity, lactic acid dehydrogenase (LDH) activity and malondialdehyde (MDA) content. LDH activity and MDA content were determined using LDH and MDA kits respectively as instructed.

### Statistics

All data were expressed as the means ± SD. An one-way ANOVA using SPSS 13.0 sofeware was used to conduct a statistical comparision of differences among the groups and a value of P < 0.05 was considered as statistically significant.

## Additional Information

**How to cite this article**: Cui, H. *et al.* Dioscorea bulbifera polysaccharide and cyclophosphamide combination enhances anti-cervical cancer effect and attenuates immunosuppression and oxidative stress in mice. *Sci. Rep.* **6**, 19185; doi: 10.1038/srep19185 (2016).

## Figures and Tables

**Table 1:** The weights of the animals of the experimental groups.

Group	Initial weight (g)	Final weight (g)	Body weight increased (%)
Blank control	18.1 ± 0.5	25.0 ± 0.9	38.1 ± 0.8**
Positive control	18.3 ± 0.6	22.8 ± 0.9	24.6 ± 0.5**
Negative control	18.1 ± 0.3	21.3 ± 0.7	17.7 ± 1.3**
Low dose group	17.9 ± 0.7	23.2 ± 0.5	29.6 ± 0.3**
High dose group	17.7 ± 0.6	23.4 ± 0.4	32.2 ± 0.3**
Low dose group + CTX	18.0 ± 0.5	24.2 ± 0.8	34.4 ± 0.6**
High dose group + CTX	18.1 ± 0.8	24.5 ± 1.1	35.4 ± 0.4**

Compared with the negative control group, **P < 0.01. CTX – cyclophosphamide.

**Table 2:** Effects of CTX treatment with or without DBLP on U14 solid tumor growth in mice.

Group	Tumor weight (g)	Inhibition rate (%)
Positive control	1.15 ± 0.50	65.26 ± 0.02**
Negative control	3.31 ± 0.49	0
Low dose group	2.46 ± 0.17	25.68 ± 0.65**
High dose group	2.07 ± 0.45	37.46 ± 0.08**
Low dose group + CTX	0.96 ± 0.18	71.00 ± 0.63**
High dose group + CTX	0.85 ± 0.19	74.32 ± 0.61**

Compared with the negative control group, **P < 0.01. CTX – cyclophosphamide.

**Table 3:** Effects of CTX treatment with or without DBLP on the percentages of peripheral blood T-lymphocyte subpopulations in tumor-bearing mice.

Group	CD^4+^ gated %	CD^8+^ gated %	CD^4+^/CD^8+^
Blank control	66.22	3.85	17.2
Negative control	70.80	3.95	17.92
Positive control	83.26	1.85	45.01
high dose DBLP	67.45	4.98	13.54
high dose DBLP + CTX	67.18	3.04	22.10

CTX – cyclophosphamide; DBLP – *Dioscorea bulbifera* polysaccharides.

**Table 4:** Experiment grouping and treatments received.

Group	1	2	3	4	5	6	7
Dstilled water (ml)^a^	0.2	0.2					
Low dose DBLP (100 mg/kg) (ml)^a^				0.2		0.2	
High dose DBLP (150 mg/kg) (ml)^a^					0.2		0.2
CTX (ml)^b^			0.2			0.2	0.2

^a^Intragastric gavage.

^b^Intraperitoneal injection; CTX – cyclophosphamide; DBLP – *Dioscorea bulbifera* polysaccharides.
